# Qualitative assessment of South African healthcare worker perspectives on an instrument-free rapid CD4 test

**DOI:** 10.1186/s12913-019-3948-x

**Published:** 2019-02-14

**Authors:** Fiona Scorgie, Yasmin Mohamed, David Anderson, Suzanne M. Crowe, Stanley Luchters, Matthew F. Chersich

**Affiliations:** 1 Wits Reproductive Health and HIV Institute (Wits RHI), Faculty of Health Sciences, University of the Witwatersrand, Johannesburg, South Africa; 2 Burnet Institute, Melbourne, Australia; 3 School of Public Health and Preventive Medicine, Monash University, Clayton, Victoria Australia; 4 Department of Population Health, Aga Khan University, Nairobi, Kenya; 5 International Centre for Reproductive Health, Department of Public Health and Primary Care, Ghent University, Gent, Belgium

**Keywords:** CD4, South Africa, HIV, Point of care, Acceptability, Qualitative research

## Abstract

**Background:**

Accurate measurement of CD4 cell counts remains an important tenet of clinical care for people living with HIV. We assessed an instrument-free point-of-care CD4 test (VISITECT® CD4) based on a lateral flow principle, which gives visual results after 40 min. The test involves five steps and categorises CD4 counts as above or below 350 cells/μL. As one component of a performance evaluation of the test, this qualitative study explored the views of healthcare workers in a large women and children’s hospital on the acceptability and feasibility of the test.

**Methods:**

Perspectives on the VISITECT® CD4 test were elicited through in-depth interviews with eight healthcare workers involved in the performance evaluation at an antenatal care facility in Johannesburg, South Africa. Audio recordings were transcribed in full and analysed thematically.

**Results:**

Healthcare providers recognised the on-going relevance of CD4 testing. All eight perceived the VISITECT® CD4 test to be predominantly user-friendly, although some felt that the need for precision and optimal concentration in performing test procedures made it more challenging to use. The greatest strength of the test was perceived to be its quick turn-around of results. There were mixed views on the semi-quantitative nature of the test results and how best to integrate this test into existing health services. Participants believed that patients in this setting would likely accept the test, given their general familiarity with other point-of-care tests.

**Conclusions:**

Overall, the VISITECT® CD4 test was acceptable to healthcare workers and those interviewed were supportive of scale-up and implementation in other antenatal care settings. Both health workers and patients will need to be oriented to the semi-quantitative nature of the test and how to interpret the results of tests.

**Electronic supplementary material:**

The online version of this article (10.1186/s12913-019-3948-x) contains supplementary material, which is available to authorized users.

## Background

From the earliest days of HIV care and treatment, CD4 cell count measurements have played a central role in the care of HIV-infected children and adults. CD4 counts are an accurate predictor of disease status and the immediate risk of death, and are thus used for identifying those who have advanced HIV disease and whose care needs to be prioritised [1]. Over the past decade, antiretroviral therapy (ART) in HIV-positive patients has been initiated at progressively higher CD4 counts [2]. World Health Organization (WHO) guidelines increased the ART initiation threshold from 200 to 350 cells/μL in 2010, to 500 cells/μL in 2013, and then in 2015, recommended treatment for all HIV-infected patients, irrespective of CD4 count [2, 3]. This significant shift in treatment protocols has unfolded against the background of ambitious UNAIDS treatment targets of “90-90-90 by 2020”, and has brought the ongoing relevance of CD4 testing into focus [4].

CD4 measurement is still included in WHO and national guidelines in South Africa and other countries as part of the battery of tests done prior to ART initiation [5–8]. In this instance, CD4 testing informs the prioritising of ART initiation in patients with a CD4 ≤ 350 cells/μL, and determines the need for opportunistic infection prophylaxis (at a CD4 count of ≤200 cells/μL) and for testing for cryptococcal antigenaemia (at a CD4 count of ≤100 cells/μL) [6]. Further, WHO recommends that, in settings where HIV viral load (VL) testing is not routinely available, a CD4 count should be done 6-monthly in patients receiving antiretroviral treatment. These test results, interpreted in conjunction with clinical monitoring and a patient’s history of drug adherence, are used to diagnose treatment failure [9]. Though VL testing is increasingly available, only about half of the estimated 20 million people receiving ART worldwide have access to it [10]. Where VL testing is available, CD4 count monitoring can be reduced in frequency or stopped, in those who are stable on ART and are virally suppressed. Patients with unstable or advanced HIV disease still require CD4 monitoring, however, even when VL testing is readily available [7].

Flow cytometric methods have been the gold standard for CD4 monitoring since the beginning of the HIV epidemic, but these methods require specialised electronic instruments, and typically need constant power or batteries, intensive quality control procedures, as well as highly-trained laboratory staff [11]. These requirements have constrained CD4 testing in a number of resource-constrained settings, but especially for rural populations [12–15]. Moreover, patients in many countries lack a unique patient number, making it very difficult to trace test results from different laboratories, especially in patients referred between hospitals and clinics. In one study in Mozambique, Malawi, and South Africa, as many as 50% of CD4 test results were not returned to the clinics [16]. Testing at the point of care (POC) may overcome many of these access barriers and enable poorly resourced and remote facilities to prioritize patients for ART eligibility and monitor their ART responses more effectively.

**Fig. 1:**
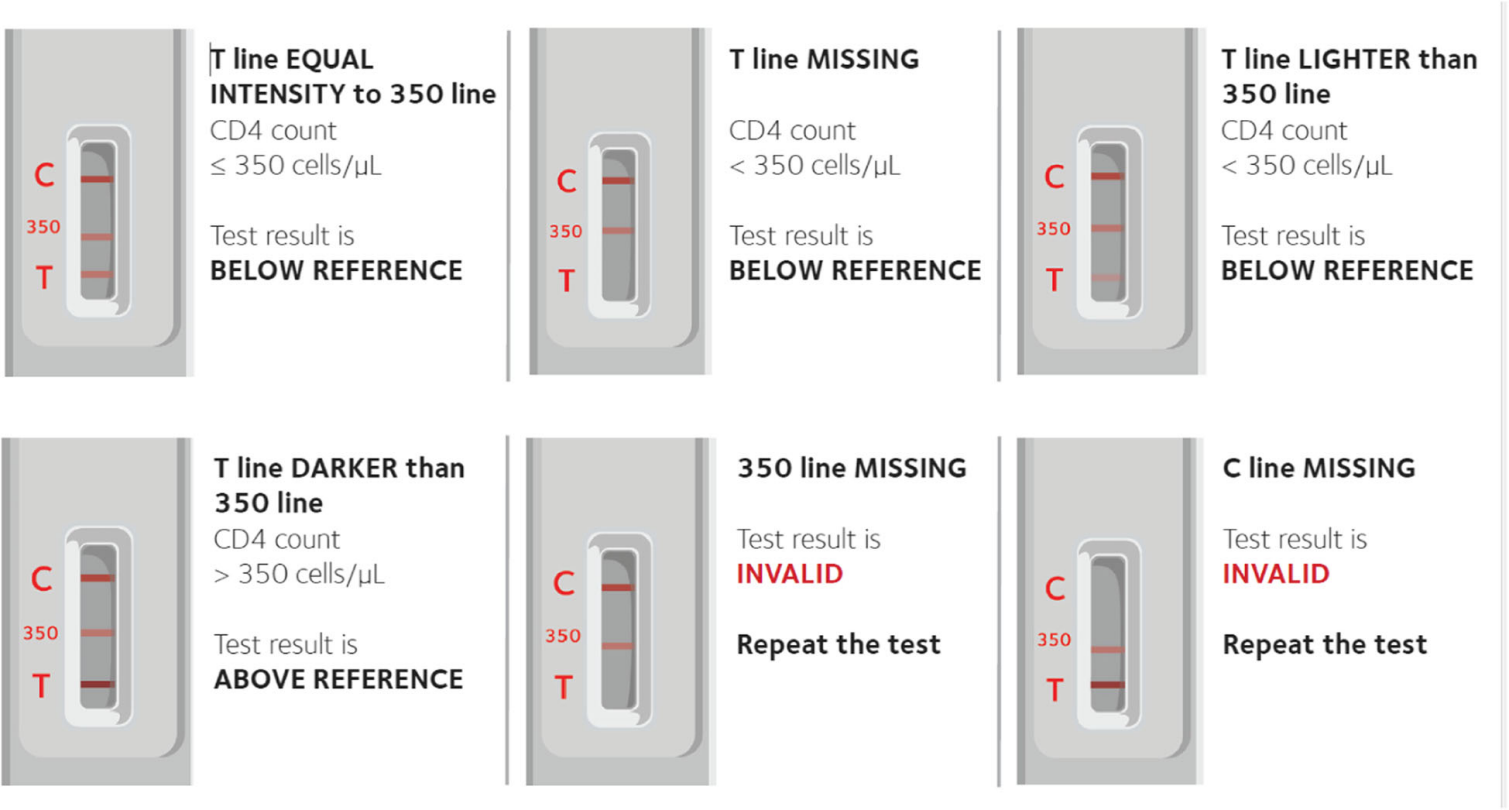
VISITECT®CD4 test strip

POC CD4 testing may also be more cost-effective than laboratory-based methods [17, 18]. It has been found to facilitate timely decision-making and care linkages, and help improve patient care and retention in most studies [12, 13, 19, 20], but not all [21, 22]. To date, all POC CD4 tests have required an instrument [23–25], thus incurring many of the limitations of machines in central laboratories, including frequent error codes, technical breakdown, and lack of access to technical support [26, 27]. The Burnet Institute has developed a POC CD4 test that can be used without an instrument (VISITECT® CD4, Omega Diagnostics, UK) [28], based on a lateral flow principle, which gives visual results after 40 min (Fig. 1) [29].

Understandably, much of the research focus around the development of CD4 diagnostics has centred on the validity of the test [12, 27, 30]. Inaccurate measures of CD4 cell count can have important consequences, especially if patients are misclassified as having treatment failure, or missed when their treatment has, in fact, failed. While test validity clearly warrants attention, factors such as end-user perspectives, effect on patient flow within the clinic, and the fit with user needs are also important [31]. However, the views of healthcare workers who perform the tests and, ultimately, of patients themselves have seldom been examined. Even more rarely are qualitative methods used in such evaluations [26, 32, 33].

We report here on a qualitative study that formed one component of a Field Testing study, in which the validity of VISITECT® CD4 was evaluated within an antenatal clinic in South Africa, and before the device obtained CE marking (November 2017) [34]. Our aim was to provide an in-depth understanding of the views of healthcare workers on the acceptability and feasibility of rapid, POC CD4 testing in this clinical setting. The findings may have broader relevance for those developing similar POC diagnostic tools, and for health communication specialists who have an interest in designing appropriate education and training materials for healthcare workers and patients alike.

## Methods

### Study setting and routine care

Rahima Moosa Mother and Child Hospital (RMMCH) is a tertiary-level public sector hospital in Johannesburg. Women attending the hospital are either locals from surrounding areas or high-risk cases referred from other antenatal clinics in the region. The HIV prevalence among women giving birth at the hospital is estimated to be in the region of 25% [35]. The programme for preventing mother-to-child transmission (PMTCT) of HIV infection is well established and has been progressively strengthened over time [36–38]. Current rates of HIV transmission from mother to infants are 1.6% [35]. CD4 cell count testing is routinely performed at the first antenatal visit for all pregnant women with HIV, and blood samples are sent to an external quality-controlled laboratory for CD4 count testing using standard flow cytometry methods. The turn-around time for results from laboratory to antenatal clinic is between 1 and 3 days. Patients only receive results when they return to the hospital for their subsequent antenatal care visit or to give birth. Viral load testing is available at the site and used to detect antiretroviral treatment failure.

### Study design

In April 2017, the perspectives of a small group of healthcare workers at RMMCH were elicited using in-depth, semi-structured interviews. These were healthcare workers who had engaged with the VISITECT® CD4 test during the Field Testing study, either in the hospital laboratory or in the antenatal clinic. Participants were interviewed individually to allow for detailed discussion of specific experiences with the test among diverse levels of health workers. Qualitative data were not collected from patients themselves as they had not been given the results of the VISITECT® CD4 test, instead only receiving the flow cytometric CD4 testing, as per standard of care.

### VISITECT® CD4 testing procedures

**Fig. 2:**
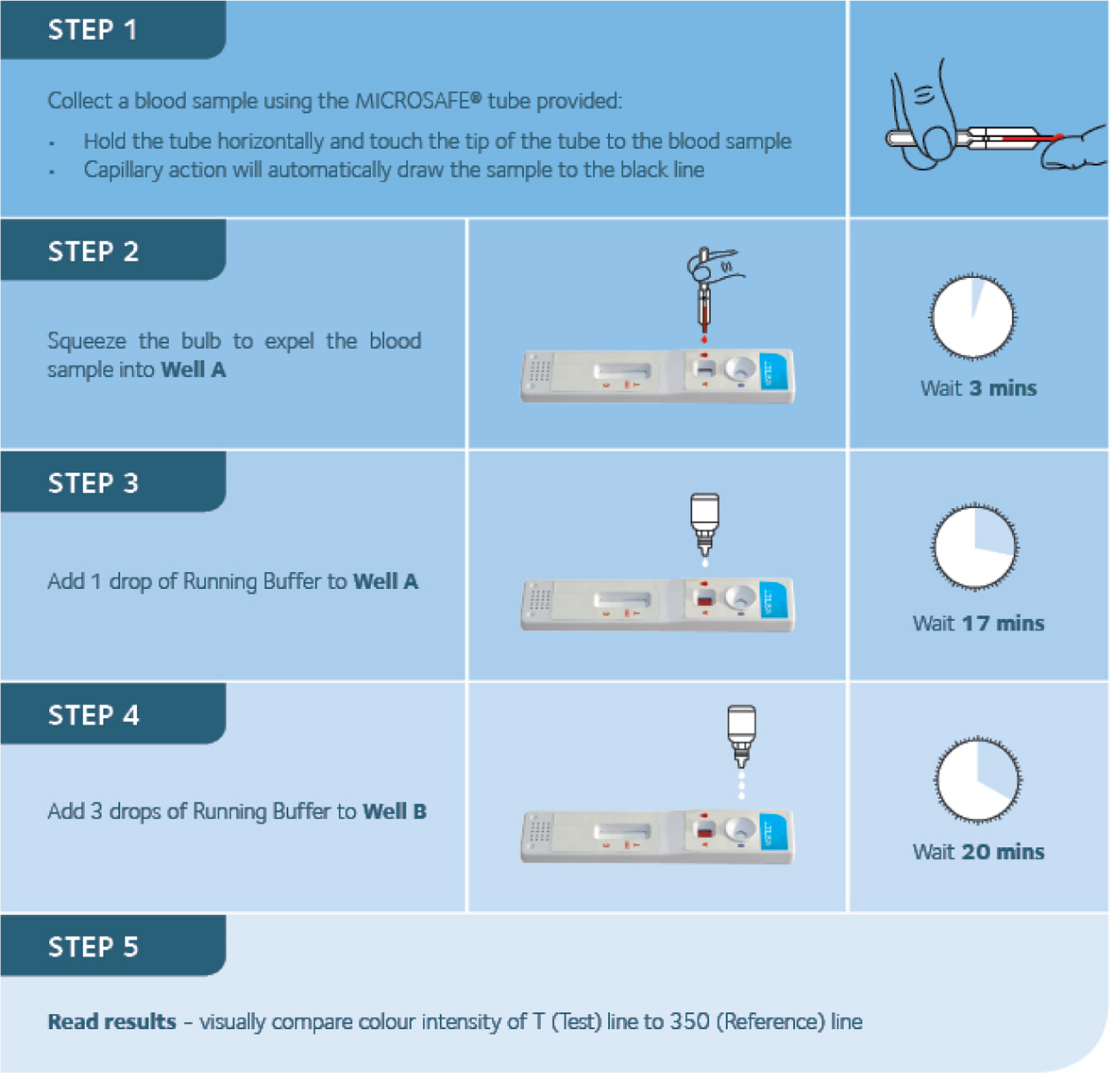
Timing and procedures for each step of the VISITECT® CD4 test

Blood samples were collected using both capillary tubes from a finger prick and by venepuncture. Venous samples were tested in the hospital laboratory as well as at the point of patient care, while finger prick samples were tested only at the point of care. In the first step of the test, 30 μL of blood was applied to the test strip, and buffer solution added to the strip after 3 min and again 17 min thereafter. The test result was read 20 min later (40 min in total; see Fig. 2).

The test strips were read both visually and with the use of a hand-held electronic reader (VISITECT® CD4 Reader). The test is semi-quantitative, with visual results in the form of a line on the strip, similar to that seen on a pregnancy test. The line indicates whether the patient’s CD4 count is above or below the set threshold of 350 cells/μL (Fig. 1). Three months prior to the study enrolment, healthcare workers attended 2 days of training on study procedures and completed practical sessions with the device. Following completion of the first 50 practice tests, a refresher training was repeated immediately prior to the onset of the study.

### Participant selection and interview

For the qualitative sub-study, all eight health workers who had been directly involved in the project were invited to participate in interviews. All agreed to be interviewed. Participants included a diverse range of healthcare workers, namely: two counsellors, three registered nurses, one medical officer, one phlebotomist and a laboratory worker. The medical officer and the three nurses were based in the hospital’s Research Unit, while the laboratory worker was stationed in a small laboratory dedicated to research activities. Both counsellors worked in the antenatal clinic and the phlebotomist was based in the postnatal ward but also collected blood samples from the antenatal clinic. All but one of the participants were female, and the number of years they had been working in their current position ranged from two to 23 years, with a median of 7.5 years.

Participants were interviewed by a member of the study team who is a qualitative social scientist (FS). All interviews took place in a private room within the hospital. A semi-structured interview guide was developed following discussion among members of the study team, which included researchers who had been involved in training for the field testing study and the product developers (Additional file 1: Interview Guide). The guide included open-ended questions on the following themes: healthcare workers’ perceptions of the role of CD4 testing; experiences of using VISITECT® CD4 and acceptability of the test; perceptions of the validity of the test; and views on obtaining semi-quantitative results. Interviews were in English and lasted between 35 min and 1 h each. All were audio-recorded.

### Data management and analysis

Audio recordings were transcribed by a transcription service and checked by the interviewer to ensure fidelity to the recordings. Using a grounded theory approach [39], the transcripts were then reviewed and inductively coded according to emerging themes. These themes were used to develop a matrix that allowed for comparison of key themes across all participant transcripts and the identification of patterns in the data [40]. Data analysis was led by the interviewer, who discussed key themes and interpretation of findings with the rest of the study team. Text for the paper was then constructed on the basis of this completed matrix. Participant quotes were selected to illustrate key findings from the analysis.

## Results

### Importance of CD4 testing

Even in the era of ‘test and treat’, all participants interviewed for the study felt that CD4 count testing remained an important diagnostic and monitoring test in this setting. They cited the need for a CD4 ‘baseline’ for patients initiating ART and they felt that ongoing CD4 monitoring was required to measure the need for OI (opportunistic infection) prophylaxis, ART response and a switch in treatment regimens.

However, the view of some participants – mainly the nurses and the medical officer – was that increasingly, VL measurements were used for monitoring patients’ progress.

*In the clinics we focus on looking at their viral load instead of the CD4 count. But, then it’s still important to check the CD4 count because… are the cells in the blood becoming strong? [Nurse].*

As the nurse’s comment suggests, these two measurements were considered complementary and in need of simultaneous appraisal. CD4 count results were also regarded as a useful marker to alert healthcare workers to possible adherence failures, even becoming vital information to *motivate* patients to adhere.

*So when she knows exactly what is going on in her body [after having a CD4 test], yes; it is a good motivation. She would want her CD4 to go up. [Nurse].*

Participants also noted that patients generally have a good understanding of CD4 testing and the role that CD4 cells play in relation to HIV. They attributed this to the substantial counselling and education that patients have received in HIV Counselling and Testing (HCT) settings and in ART clinics in recent years. Consequently, many patients have a strong desire to know their CD4 count results.

*The only thing that they are worried about is the CD4 count, because everybody has been saying, CD4 count, CD4 count… [Counsellor].*

### Acceptability of rapid tests for CD4 testing

#### User-friendliness

Overall, the VISITECT® CD4 was regarded by all healthcare workers interviewed as an “easy” test to perform, generating results that were “easy” to read. Both the laboratory worker and the phlebotomist emphasised the “straightforwardness” of VISITECT® CD4 test procedures, and their familiarity given prior experience in using other rapid POC tests that require finger pricks, such as the rapid pregnancy test and rapid HIV antibody tests.

*So, if you know how to do the reading visually, then I don’t think there should be a problem. If it’s accurate, then it will be a pleasure to work with this kind of test. It’s straightforward, it’s easy, it’s not time consuming. [Laboratory Worker].*

After further probing about specific aspects of the test, participants drew attention to what they had experienced as a very small margin for error in performing it. As they described it, the set-up was quite unforgiving in its demand for precision in each of the test’s five segments or steps (Fig. 2), which made it less user-friendly as a result.

*There seems to be an absolute need to run this perfectly, in the lab when you run the test, as in not 35* μl*, not 25 μl, but 30 μl of blood. And so who knows what, with the mixing and all the little things… The time has to be exact... No, I mean if you’re not dedicated to it and you come a few minutes late because you’re busy with something else... it makes one worry. [Medical Officer].*

In practice, this meant that those performing the test needed focused concentration and minimal interruptions to avoid errors or miscalculations in the timing and completion of each step. Participants who had some experience in collecting blood samples and applying them to the test strips advised that it was essential to develop a “system” to ensure correct performance of each of the five steps. A nurse phrased this as needing to “structure it nicely”, while a counsellor stressed it was important to shut out other distractions and “be focussed”. Importantly, the time lag between some steps, as much as 20 min between steps 4 and 5, meant that most healthcare workers tried to multi-task and make productive use of this wait. These attempts were only partially successful: some found the multi-tasking too distracting, thus endangering their ability to return to the test strip in time. One participant found this need for precision to be stressful and believed that, if done at the ‘bedside’, it could interfere with the quality of counselling.

*… you are losing focus here and you don’t want to leave this patient unattended as well, because there are things that she needs to know, maybe about, regarding the CD4 count, the viral load, and once you neglect the patient as well, it’s a problem. [Counsellor].*

To some extent, perceptions of user-friendliness depended on where in the facility the test would be positioned and who would actually be responsible for performing it. Those who found it challenging to multi-task within a clinical environment believed that the test was best handled in the laboratory. Alternatively, to resolve the dilemma of how to perform a test that requires concentration and focus, while at the same time managing consultations with individual patients and juggling other administrative duties, three participants felt that a dedicated staff member should be tasked with performing the POC CD4 test.

*If there’s one person who is allocated to do only this job then it could work. Even with the nurses, you know, I don’t see every nurse who sees a patient doing the test. So there should be one person who has that role, they do the test and they focus only on the test. Also, it will minimise problems of errors happening. [Nurse].*

Those who were not troubled by this need for focus and precision tended to rely on two items that served as *aides-memoire*: a timer with an alarm to signal the end of each time segment, and a wall chart depicting the sequence and duration of each step. One nurse described how these tools were used:

*Somebody will be coming in… and I say okay wait, okay, I need to set my timer, I’m waiting for the 17 min to end so that I can set up the next one. So, before that, I cannot move from here. It’s a fairly easy test to run. You just need to make sure that you’ve got your timer… You always need to have that guide on the wall and keep checking, keep checking. That helps. [Nurse].*

One counsellor was puzzled by why multiple steps were needed in the testing procedure at all, and called for these to be reduced or collapsed into a single step:

*The need of improvement is the time, because I am really not sure why are we waiting 3 min and then the 17 min and then the 20, I don’t know why that is happening [….] You know? Instead of doing the thing at the same time, and see what it’s going to give us. [Counsellor].*

Closely related to the question of ease-of-use is the concern about whether a new task added to a healthcare worker’s scope of work would be perceived or experienced as a workload burden – and therefore risk generating resistance to the task. Participants were asked to imagine the VISITECT® CD4 test being added to their current scope of work: what would that mean for them, personally?

Overall, the responses were positive and the handling of rapid CD4 count tests was not seen as necessarily implying an added burden to workloads. One of the counsellors was cautious, however, remarking that “it will depend on how many people we are seeing”, while another counsellor considered the extra task manageable only if you were “organised”.

Related to the question of scopes of work, participants were asked which level of healthcare worker would be best placed to administer the VISITECT® CD4 test, should it be integrated into routine care in antenatal settings. Assuming the blood sample would be drawn from a finger-prick rather than by venepuncture, two of the nurses believed that administrating the test would fit well within a counsellor’s scope of work and skill-level. As one put it,

*I don’t foresee any problems if the counsellor actually does the whole process, but it will mean proper supervision and making sure that every step goes very well. [Nurse].*

This idea was supported by the counsellors themselves. Citing the unique focus and skills of counsellors working in an environment with many HIV-positive patients, they claimed the test could serve to further extend the patient-centred care they provide:

*I think the counsellor is the best person to do that, because already they are going to have that relationship, they are going to have that interaction, they would have spoken about the CD4 count as well… you are talking about treatment, you are talking about the viral load and everything. So you would have aligned everything, and then you are going to say now you are going to do this [test], okay. [Counsellor].*

#### Turn-around time

Presently, counsellors in the ANC clinic record the names of all patients awaiting CD4 count results in a register as part of standard care, and then follow up a few days later to record the results as they are received from the central laboratory. The relevant healthcare workers review these results as a team, and a decision is made about which results are concerning enough to warrant the patient being called back to the clinic earlier than their next scheduled visit. These patients are then phoned and requested to return to the clinic promptly.

The counsellor who explained this process maintained that, “for me, it’s not working”. The time lag between test administration and conveying of results to the patient, she felt, was too long. Furthermore, some patients never received their CD4 count results because they did not return to the facility, possibly because they feared that these results might signal deteriorating health.

In theory, the introduction of POC CD4 testing would alleviate this concern, eliminating the need for counsellors to follow-up and check that all results had been returned from the laboratory. Undoubtedly, participants saw this as the greatest strength of the test: CD4 count results could be relayed to the patient immediately and decisions about follow-up treatment be made there and then. The test was regarded as valuable in terms of improving continuity of care, but cost savings were also mentioned as a positive off-spin.

*Another thing that I would like to add on, mina [me], I would just say a big “up” to this test, we can use it, it’s going to save us money, it’s going to save the patient a trip, it’s going to save the patient a whole lot of things, you know. [Counsellor].*

A number of participants believed the time-saving dimension of the VISITECT® CD4 test would help in particular to alleviate patients’ anxieties about their health.

*Most of the patients that I have come across, they become so worried – ‘I think I am getting sick and maybe my CD4 count has dropped’. So with that giving them their results immediately and with them knowing whereabouts, it really makes a lot of difference […] unlike having to wait for the next visit. [Nurse].*

But a quick turn-around had benefits also for healthcare workers themselves. One counsellor pointed out that the absence of an immediate CD4 result made it harder for them to offer tailor-made counselling to the patient. She described the impact of such efficiencies: “it will make me feel great, that this woman is going to go home knowing exactly what is happening with her”.

Similarly, a nurse recounted how a doctor (not involved in the study) had witnessed her administering a VISITECT® CD4 test and had asked what she was doing. The nurse had explained the study and how the VISITECT® CD4 test kit worked.

*And she [the doctor] said ‘This would be great when we have our patients in front of us, and we need an indication now, of where the patient is… ‘Okay, let’s do a CD4, quickly and let’s see where you are.’ [Nurse].*

Overall, most participants felt that adding a POC CD4 cell count test to their repertoire of monitoring tools would strengthen the package of care currently offered to patients.

*They [mothers] would be happy to know when they leave, this is my CD4 count, ... because you always tell them during counselling that we don’t only want your baby to be alive, but we want you to be around for your baby. So it actually gives us a* *full package* *when taking care of the mother and taking care of the baby. So to me, that is a huge advantage. [Phlebotomist; emphasis added].*

All were supportive of the VISITECT® CD4 test being scaled up and implemented in antenatal settings around the country, once fully approved. Implementation was recommended, in particular, in large antenatal clinics and labour wards where there were likely to be many patients who had not attended antenatal care and thus had an unknown HIV status and CD4 count. Participants also saw a role for the test in “normal routine clinics, where they are doing the rapid tests, HIV tests, [and] in the mobile clinics” [Counsellor], as well as “in ARV clinics” [Nurse]. Health facilities in especially resource-poor areas and those located some distance from large urban centres were identified as sites where rapid CD4 count testing would be particularly useful.

*Especially in... the rural areas, bloods get sent away. Many times, bloods get lost in transit or when it goes to the main lab, there’s always the human error sometimes, wrong hospital numbers, wrong names, you know the results are there but they just can’t find it. So I think this [VISITECT® CD4] will be useful. Definitely. [Laboratory Worker].*

#### Reliability and accuracy

Study participants were given the hypothetical options of 70 and 90% accuracy and asked which level they considered to be ‘good enough’. Most regarded 90% as the minimum level acceptable:

*Well, nothing is 100% accurate [laughs], but I would still go for it, because I feel that it is... the HIV test and all the other tests that use the same process, I don’t think they are like 100% most of the time, so if it’s 90%, it’s good enough. [Phlebotomist].*

In an attempt to put accuracy issues in context, the medical officer emphasised the importance of the test not missing “the low ones” – patients with very low CD4 counts, who needed urgent intervention. Linked to this, he raised the potential for the test to serve as a screening mechanism, where, for example, patients who registered a CD4 count below 350 would go on to have a confirmatory flow cytometry test in the laboratory.

Although explored only indirectly, the interviews did address the question of whether healthcare workers thought patients themselves would trust the results of rapid POC tests. Participants thought some patients preferred tests to be performed in front of them, rather than having the sample sent away to a laboratory, where errors could happen and patients would be none the wiser.

*Patients are used to this [rapid test], unlike something you don’t see, when it goes to the lab and when it comes back, you can always think, maybe they made a mistake. But if you’re there, you can see it happening. [Phlebotomist].*

Generally, health workers believed that all POC tests – not only VISITECT® CD4 – were vulnerable to questions about accuracy. Where inaccuracies arose, there were obvious consequences for patients, but also for healthcare workers. Several participants made it clear that they did not want to work with unreliable tests, which they believed would undermine their confidence in the workplace.

*I wouldn’t have the confidence to be saying what I’m saying, you know, I would be mumbling and… then I would be hoping that there isn’t that person who would be asking me, you know, difficult questions…because if I am in that scenario I would not be able to tell false information; I’ve got to say it as it is. And if I’m looking at you and I’m saying this test is not reliable, I mean, you know... [Nurse].*

In short, there was a strong emphasis on the importance of healthcare workers being able to trust the diagnostic tools they were using.

#### Eye versus machine: Views on the VISITECT® CD4 reader

There were mixed views on whether a visual read-out of the test result was more or less reliable than obtaining a reading from the CD4 Reader that was used in the Field Testing study. The laboratory worker – who, out of all participants, had the most experience of using both methods – had this to say of the CD4 Reader:

*The instrument itself, it’s such a small machine, and it’s very user-friendly. The machine should tell you what to do, what the next step is. So I think it’s a great machine. And I would go for this in the lab. [Laboratory Worker].*

The majority were adamant that the Reader generated the most reliable results, even if they did not have direct experience of using it. This belief seemed to come from a deep-seated trust in the credibility of electronic technology in general.

*I think we now have a reading eye, we do now have a reading eye. [laughs] But I will believe the machine… I think the machine is less stressful, because with the eye… you’re reading and reading and you think, ‘it’s even’. If it’s even it might be below? …So, I won’t trust the eye. [Counsellor].*

This reference to a “reading eye” came up in other interviews, where participants spoke of the confidence in doing visual read-outs that develops with experience. About a third of the participants claimed they actually trusted their visual interpretation of the test strip more than the Reader-generated result, one saying:

*On the strip it will be clear that it’s below or it’s above. But then when you put it in the reader it gives you something else. It’s not all the time. So now I said no, you know what? My eyes are not playing tricks on me. It is that high, so it’s above. I’m sticking to that. [Nurse].*

#### Views on the semi-quantitative nature of VISITECT® CD4

Participants were divided on the acceptability of the semi-quantitative nature of the VISITECT® CD4 test (where the result is ‘above’ or ‘below’ a certain threshold, rather than an exact number). One disadvantage of the semi-quantitative calibration, according to most participants, was that it did not allow for ‘borderline’ cases to be distinguished. The laboratory worker explained the problem thus:

*If my machine tells me I’m above 350, what if it’s just borderline, where it’s* *just* *350? And if it tells me I’m below 350, what if it’s 349? …So I still think that if we could get an exact reading, that would be great, you know? … We should know the exact value. [Laboratory Worker].*

Similarly, a semi-quantitative test could not tell you *how far* below or above the cut-off level a patient’s CD4 count was. As a nurse explained,

*I think it’s a problem because now, you know, it says below 350 so you could be 340 or you could be 20… So it doesn’t really give you a feel of where is this patient. I want the numbers. [Nurse].*

Participants were quick to distinguish between their own preferences on this issue and those of their patients. Most indicated that – as healthcare workers – they did not necessarily need an exact number. One nurse described how she conveyed and explained CD4 count results to her patients by referring to a wall chart that allowed her to pinpoint the result in relation to the 350 cells/mm^3^ threshold:

*What’s important is being above 350. They’re going to pray to be above 350. And even though, I mean, you’re going to show them. This is the line. And your CD4, it’s above…So, if we’re giving them results, then we can say, you know, this is your line, this is where you fall. On that chart. [Nurse].*

Similarly, several participants said that when communicating CD4 count results to patients, they tended to use only the terms “high” or “low”, even when exact numbers were available. Most, however, recognised that a more precise measurement of CD4 count was at times important for patients as it helped them to monitor and understand their progress more accurately. In other words, one could be told that one’s CD4 count was “above 350”, but some patients then wanted to know: how *far* above 350? Receiving an exact number.

*…gave them that satisfaction to know that they are doing very well. So knowing the value actually makes a big difference. [Nurse].*

Finally, there was even a suggestion that a semi-quantitative test had some *advantages* over a purely quantitative test, as over time it could help to lessen patients’ ‘fixation’ with a number and the anxiety that inevitably arose when that number changed, even marginally.

*With the CD4 count, people are used to getting a number and a clear CD4 count. People will still ask, you know, “my CD4 dropped from 800 to 600 and what does that mean?” and they get anxious about that, start to worry a lot about it. So it [a semi-quantitative test] might reduce some of those anxieties…in that it doesn’t get people too worried about the level change. [Medical Officer].*

### Communicating test procedures and results to patients

Participants were asked to consider how they would actually communicate the purpose of the VISITECT® CD4 test, and the less-than-100% accuracy of the results in particular, to their patients. Again, the visual similarity between the VISITECT® CD4 test-kit and other POC tests – such as the rapid HIV test – made this task much easier.

*One thing I like about this kit, is it’s familiar to them. Because they know the strips – because of the HIV [rapid antibody] test. [Phlebotomist].*

One participant suggested that future implementation of the VISITECT® CD4 test in clinical settings might benefit from being accompanied by ‘job aids’ – like flip charts – that make use of visual diagrams and illustrations to communicate complex concepts to patients.

*I think what would be great for this study, if it gets implemented, is having diagrams. Simple diagrams, with, like, I draw Xs and Os in my blood vessels. So the Xs are the viral load and the Os are the CD4. And if you take your treatment, this is what it will look like. And you know, your CD4 has to look like this, then. Simple things… Just because people are better off if you show them. [Nurse].*

Participants were unanimous in believing that patients should be made aware of the fact that results from a CD4 POC test could never be 100% accurate. Various approaches to communicating this to patients were favoured. One suggested explaining that what was more important than perfect diagnostic accuracy was the clinical judgement applied by healthcare workers, which involved ‘erring on the side of caution’ anyway in order to protect patients’ health:

*….the general sense of hey, this [test] shows us this, but it’s not 100%. But if it shows lower, chances of it being lower are high and we’d rather give you some protection [prophylaxis]. [Medical Officer].*

Others framed the openness about test accuracy in terms of the responsibility to be accountable to patients.

*I think it’s important, in case… the test did go wrong somewhere, we’re able to go back to them with a straight face and say, you know, like we said, there was a 90% chance that it was accurate, we found that there was fault with it. But it would be easier to say that for accountability reasons. [Phlebotomist].*

Finally, there was one suggestion that – should the test be administered by counsellors or nurses as part of their battery of routine tests performed on antenatal patients – it could actually be viewed as an opportunity to improve patient care in somewhat creative ways. It was the hope of the medical officer that the additional time needed in each patient consultation to administer the rapid CD4 count test and wait for results could be used to improve the quality and quantity of counselling offered to a patient, that it would become.

*…part of the proper understanding of patients and not just an extra burden which will make the sisters more frustrated and less empathetic…. And so it can be done in that context of spending enough time with people… I guess it doesn’t really solve the issue of levels of communication and quality of communication to the patients, but…hopefully one would study it further and link it with that element, not just doing a technical thing. [Medical Officer].*

## Discussion

Findings from our study address a key gap in evidence as few studies have applied qualitative methods to examine the acceptability and feasibility of POC CD4 testing among healthcare workers [26]. Although use of CD4 count measurements to determine ART eligibility has declined in a number of countries, CD4 tests remain important for triaging care at the time of HIV diagnosis and thereafter [3]. In this qualitative study of the perspectives of healthcare workers in a South African antenatal setting, CD4 cell count testing was considered to have continued relevance, most notably for establishing a ‘baseline’ for patients initiating ART, monitoring treatment progress and overall patient wellbeing. In assessing the VISITECT® CD4 test, participants considered its greatest strength to be its quick turnaround of results and cost-saving potential, both for patients and health services. This has been reported elsewhere in relation to POC CD4 testing more generally [41]. The quick turn-around of results could expedite clinical decisions, particularly about the initiation of OI prophylaxis [42]. This is especially important for specific groups of patients with high loss-to-follow-up rates. In this hospital setting, patients referred from other facilities with incomplete medical records were often lost to follow-up, as were patients from outside South Africa presenting for childbirth without having attended antenatal care.

Participants in our study also viewed the VISITECT® CD4 test kit as ‘user-friendly’ and easy to use, at least partly because of their familiarity with lateral flow technology, from using other POC tests, such as rapid HIV antibody and pregnancy tests. VISITECT® CD4’s rapid POC, instrument-free technology for measuring CD4 cell counts is therefore ideally placed for use in diverse healthcare settings, including rural or remote areas and in primary care facilities or community outreach, such as in mobile clinics and other forms of community or household campaigns [41, 43, 44].

The additional workload of performing rapid CD4 testing at point-of-care, in the absence of financial incentives or additional staffing, has been identified as a major barrier to its introduction in clinical settings in LMICs [26, 45]. Participants in our study revealed nuanced views on the question of additional workloads. Overall, they did not regard adding the VISITECT® CD4 test to their scope of work as necessarily burdensome, but expressed concern about the demand for precision in performing the five steps of the testing procedure. Concentration and avoidance of interruptions were necessary to avoid errors, and some anticipated that this would impact negatively on patient care. It was suggested that additional research be done to explore whether the extra time required for the test in each patient consultation could be used to deepen counsellors’ rapport with patients and improve the quality of counselling. Future guidelines will need to clarify the recommended location within health facilities and the cadre of healthcare worker most appropriate for conducting the test; failure to do so may undermine test uptake [32].

It is possible that the VISITECT® CD4 assay is more suited to implementation in laboratory settings (at least in health facilities equipped with a laboratory) than at the ‘bedside’. Conducting the testing in an on-site laboratory would not require a remote central laboratory or highly specialised staff, and might address other healthcare worker concerns about the need for precision and concentration in administering the test [31, 41]. Testing away from the ‘bedside’ could, however, potentially erode some of the cost and time benefits of the test. An alternative to relegating the test to the laboratory would be to assign full responsibility for rapid CD4 count testing (possibly along with other rapid POC tests) to a cluster of dedicated healthcare workers. This strategy might also improve test accuracy, given that performance of test procedures and reading of results may improve with practice. In our study, counsellors were widely regarded as being most suited to perform the test and have successfully performed POC testing elsewhere [46, 47].

Healthcare worker or patient preferences for finger pricks over venous sampling would favour ‘bedside’ testing. Interestingly, evidence of patients’ preferences for finger prick collection as opposed to venepuncture is mixed. One study showed that patients preferred finger pricks mainly due to continued bleeding after venepuncture [48] and because with finger pricks there was “no suspicion that blood was used for other purposes” [49]. In another study, patients favoured venepuncture due to concerns about the need for multiple pricks if sufficient volume is not obtained in one prick [50]. Health workers appear to generally favour venepuncture [26].

While there were mixed views on whether a visual read-out of the test result was more reliable than a reading from the CD4 Reader, healthcare workers interviewed in our study were unambiguous in their disavowal of tests with sub-optimal accuracy. In time, they might consider such tests more analogous to cervical cancer screening tests, such as Pap smears, which need to be confirmed with more accurate, specialised testing [51]. Communicating results when the test in question has a relatively low sensitivity or specificity would be difficult for healthcare workers. For patients receiving such results, comprehension might be equally challenging.

Views on the semi-quantitative nature of the VISITECT® CD4 assay were also mixed, but broadly, this was considered acceptable. The limitations of a semi-quantitative measure were most evident in patients with ‘borderline’ cases [48], and for determining the direction of a patient’s progress or deterioration *within* the categories of ‘above’ or ‘below’ 350. For patients, an ‘exact number’ rather than a binary higher or lower reading might assist them to monitor and understand their progress more precisely. Implementation of the test could be supported by pictorial job aids (such as flip-charts) that help health workers to explain the meaning of CD4 counts and communicate VISITECT® CD4 test results to patients [32]. Giving patients written educational information after POC CD4 testing has been shown to raise retention in care in a trial in South Africa [52].

The study has some limitations. Health workers were assessing a test whose validity had not been ascertained at the time of the study (CE marking obtained November 2017) [34]. The interviews, however, preceded as if the device would work and be used in future. Participants, when formulating their response, were asked to imagine that the test was actually being used in routine care. Nevertheless, doubts about the test’s validity may have influenced their perceptions of the test and its potential contribution to patient care. Participants’ perspectives were based on having performed a limited number of tests (an average of 150 each) and the study was unable to measure whether their views would have evolved over time as additional tests were done, or conducted as part of routine patient care, as opposed to within a discrete study in a research setting. Related to this, the healthcare workers we interviewed were research staff rather than public sector workers, implying that their views on workload need to be appraised with this in mind. Also, while assessing health workers’ perceptions of POC CD4 testing adds to the existing body of evidence, a more comprehensive ‘end-user’ assessment would be valuable for capturing the perspectives of patients and their understandings of the test, especially its semi-quantitative nature.

## Conclusions

In conclusion, assessing the CD4 cell count level following diagnosis enables triaging of HIV disease stage, and could improve ART initiation and retention rates [41, 43]. Patients with CD4 > 350 cells/μL may have improved longer-term ART adherence if they undergo regular counselling until ART readiness is confirmed [53]. The instrument-free, point-of-care VISITECT® CD4 test can be useful as a triaging device, allowing for the prioritization of patients, both in facility- and community-based care settings. This may be particularly useful in more remote settings with limited or no access to laboratory-based testing where CD4-based ART prioritization is still taking place. The device may also have a role in the monitoring of a patient’s response to ART.

The diverse range of health workers participating in this study were all supportive of the VISITECT® CD4 test being scaled up and implemented in antenatal settings around the country, once fully approved. Overall, the VISITECT® CD4 test was acceptable to healthcare workers, but questions remain about the optimum site within health facilities for testing and how best to incorporate the test into healthcare workers’ existing scopes of work. As the test is introduced in clinical settings, healthcare workers will need to be oriented to how to explain and understand rapid tests whose results have some measure of uncertainty.

## Additional file

Additional file 1: Interview Guide. In-depth Interview Guide for Healthcare Workers, VISITECT® CD4 Test Qualitative Study. This guide sets out core questions to be asked in the interview with participating healthcare workers in the VISITECT® CD4 Test Qualitative study, together with suggested probes to encourage further discussion on the main interview topics. (DOCX 32 kb)

## Abbreviations

ART: Antiretroviral treatment
ARV: Antiretroviral
POC: Point of care
VL: Viral load
WHO: World Health Organisation
