# Supplementary material for: Qualitative assessment of South African healthcare worker perspectives on an instrument-free rapid CD4 test
Source: BMC Health Serv Res. 2019 Feb 14;19:123. doi: 10.1186/s12913-019-3948-x (PMC6376755; doi:10.1186/s12913-019-3948-x)
Supplement: Supplementary file 1 — Interview Guide. In-depth Interview Guide for Healthcare Workers, VISITECT® CD4 Test Qualitative Study. This guide sets out core questions to be asked in the interview with participating healthcare workers in the VISITECT® CD4 Test Qualitative study, together with suggested probes to encourage further discussion on the main interview topics. (DOCX 32 kb) [file 12913_2019_3948_MOESM1_ESM.docx]

**IN DEPTH INTERVIEW GUIDE FOR HEALTH CARE WORKERS**

| **Interviewer Identification Number:**  ***LABEL THIS ID NUMBER ON AUDIOTAPE*** |  |
| --- | --- |
| **Interviewer name:** |  |
| **Note taker:** |  |
| **Date of Interviewer (yyyy/mm/dd)** | **______ / ______ / ______** |
| **Start Time (hh 24 hour clock/mm)** |  |
| **Stop Time (hh 24 hour clock/mm)** |  |
| **Data Check Performed by** |  |
| **Data Entry Date (yyyy/mm/dd)** | **______ / ______ / ______** |
| **BACKGROUND CHARACTERISTICS (to be completed for all participants)** | |
| **Job Title** |  |
| **Number of years working in current job** | ______ Years |
| **Age** | ______ Years |
| **Gender** | 1 Male 2 Female |

**Introduction and Description of Project:**

Hello, and thank you for agreeing to participate in this in depth interview. My name is ______________.

The purpose of this in-depth interview is to learn about your ideas and opinions on the Visitect® rapid CD4 test. Today, we will explore your experiences with explaining the CD4 test to women and with doing the actual test. We will be discussing the ways in which the test worked well and not so well. We will also talk about the advantages and disadvantages of having a test which gives a result the same day. The interview will last approximately one hour.

**Interview Guide**

- Could you tell me what role you’ve played in the Visitect CD4 POC study?
- What is your role or position more generally within the hospital?

| **Lead Questions** | **Secondary questions and probes:** |
| --- | --- |
| **Knowledge of health workers and patients about CD4 cells** | |
| Do you think that **pregnant women** understand what a CD4 cell count means? | Do you think patients understand what the different levels of CD4 cell counts mean?  In which areas are patient’s knowledge on this topic weak and need to be focused on during counselling? |
| How do you **explain** the different amounts or levels of CD4 cells to **patients** and what these different levels mean? | What they would explain to a patient with a count of 50, 340 and 1000.  As ART is widely available now and works so well, does it really matter for a patient if their CD4 count is 50 or 340? |
| Which aspects of knowledge about CD4 cell counts or CD4 testing need to be **strengthened in staff** involved in HIV counselling or care to raise their knowledge of this topic? | In which areas are health workers knowledge on this topic weak? |
| **Acceptability of same day results for women, counsellors** | |
| What are the **advantages** of doing the CD4 cell count test on the **same day** as HIV testing? | Compare this to the way CD4 count testing is done currently? |
| What are the **disadvantages** of doing the CD4 test on the **same day** as HIV testing? |  |
| **Discussion of the way test is explained to patients and done in laboratory** | |
| Can you think of any **problems** you had with explaining the way the Visitect rapid CD4 test works to patients? | Probe for any things that were easy to explain to patients, or easier than anticipated to explain. |
| Can you please talk about any **problems** you had with **actually doing** the Visitect test? | In what ways was the test easy to do?  How did using the test impact on your interaction with patients and the routine of tasks you need to get through with each patient? |
| If you think of how the test was done, can you think of anything that might be done to make the test **easier** to use, or better in anyway? | Where there any aspects of the test that you felt needed to be improved? |
| Recall there were **three ways of doing the test**:   - with the test strip placed in the machine, - with the test done using blood taken from patients as part of routine care - and the test done with a finger prick.   Which of the three ways of doing the test was **easiest** to do? | Which of the three ways of doing the test did you prefer and why? |

| **Perceptions of the validity of a Visitect® rapid CD4 test** | |
| --- | --- |
| Which of these three ways above do you think gives the most **accurate** result, and why? | Probe for any other factors they think might affect the validity of the test results. And which was the least valid method. |
| **How staff explain uncertainty around the validity of the Visitect test** | |
| *Ask staff to imagine that the test was being used in routine care.*  What do you think are the negative and positive consequences of telling patients their CD4 count is **below 350, while it is actually above 350**? |  |
| What do you think are the negative and positive consequences of telling patients their CD4 count is **above 350, while it is actually below 350**? |  |
| If the test is only **90% accurate**, how will that influence your views on the test and what you say to patients when providing the test result? | And if it is 70% accurate, how would your views change? Is there a percent accuracy at which you would say the test should not be used? |
| Would you attempt to explain to patients that you are not 100% certain if the result is correct?  If so how would you explain it? | If the test was 90% accurate, do you think we should tell patients that we are not 100% sure of the result, or should we just give them the result? |
| Imagine one day when the Visitect® rapid CD4 test is approved for use, and during routine care with its use, the Visitect test showed one result and the standard CD4 laboratory machine test showed a quite different result, what would you **tell the patient**?  Which result would **you** believe? |  |
| **Semi-quantitative test versus a quantitative test** | |
| As you know, other types of CD4 cell tests report an exact cell count, rather than just saying the cells are above or below 350. Do you think that it is important for patients to know **exactly** how many cells they have? | Think of anaemia treatment, we tell patients they are anaemic, if they have a haemoglobin under 11, and treat for anaemia. Do you usually explain to patients that their haemoglobin is 9,6, for example, or just say they are anaemic. |

Are there other settings besides this one (ANC, post-partum) where this kind of test would be valuable?

Anything else you would like to add, not already covered?
